# Adjuvant therapies in advanced hepatocellular carcinoma: moving forward from the STORM

**DOI:** 10.1186/s13063-016-1675-8

**Published:** 2016-11-25

**Authors:** Mohamed Bouattour, Olivier Soubrane, Armand de Gramont, Sandrine Faivre

**Affiliations:** 1 Department of Hepatology, Beaujon University Hospital, 100 boulevard du Général Leclerc, Clichy, 92110 France; 2 Surgery and Liver Transplant Department, Beaujon University Hospital, Clichy, France; 3 AFR-Oncology, Boulogne-Billancourt, France; 4 Medical Oncology Department, Beaujon University Hospital, Clichy, France

**Keywords:** Antiangiogenic agents, Hepatectomy, Local ablation, Clinical trials

## Abstract

**Abstract:**

Like other previous treatments and approaches, sorafenib, an antiangiogenic drug, failed to show any benefit in the adjuvant setting for hepatocellular carcinoma in a large clinical trial. We discuss reasons and implications of these negative results and the implications for clinical practice and future research.

**Trial registration:**

ClinicalTrials.gov: NCT00692770. Registered 5 June 2008. This study has been completed.

## Main text

The prevention and the delay of relapse of hepatocellular carcinoma (HCC) after curative treatment remain a major challenge. Recently the sorafenib as adjuvant treatment in the prevention of recurrence of hepatocellular carcinoma (STORM) study reported in *Lancet Oncology* by Bruix and colleagues [1] investigated whether sorafenib is an effective adjuvant treatment after resection or ablation in patients with early HCC. The study postulated a benefit of sorafenib as adjuvant treatment in the prevention of tumor recurrence and, therefore, its ability to increase patient survival. Both the primary endpoint — recurrence-free survival — and secondary endpoints — time to recurrence and overall survival — were not reached. Despite being negative, we believe that the study has several positive implications for clinical research.

First, it has important implications for the design of future adjuvant trials in HCC and for the choice of the drug to be tested. The goal of adjuvant treatment is to reach for a cure. When tumors are emerging from a pro-tumoral underlying disease, decreasing the risk of both recurrence and de novo tumors is an additional challenge. Whether these two goals are achievable with a unique drug and strategy is an open question in HCC. Assuming side effects for a limited period of time, most active treatments should effectively target tumor residues. In contrast, long-term preventive treatment of the underlying disease would require drugs with a very good tolerance profile fitting chronic administration. The latter is a challenge in the drug development paradigm in which molecules are developed for maximum efficacy in the advanced setting. However, as illustrated by this trial and others, these results are quite often not translatable to the adjuvant setting. Undetectable tumors may exist as vascularized micrometastases, non-vascularized clusters of tumor cells, and isolated or even circulating tumor cells [2]. Whereas the benefit of an antiangiogenic agent on residual vascularized tumors may be obvious, it is clearly speculative for the other tumor cell populations that lack proper evaluation models [3]. Unlike bevacizumab, a specific anti vascular endothelial growth factor A (VEGF-A) antibody, sorafenib has antiangiogenesis and antiproliferative proprieties [4]. It could be expected that sorafenib will be more potent to block residual or metastatic tumor cells independently of its antiangiogenic action. In addition, sorafenib could prevent the growth and progression of micrometastases by inhibiting de novo angiogenesis. Nevertheless, no robust preclinical or clinical data have yet confirmed such hypotheses, especially in a very heterogeneous tumor such as HCC.

In the STORM trial, no beneficial or detrimental effect of sorafenib has been reported besides a trend towards increased time to recurrence, which may reflect a positive impact of sorafenib on tumor relapse and/or prevention of de novo tumors. Investigating post-recurrence treatment strategies and outcomes may also yield valuable information in the perspective of global HCC management.

Second, adjuvant strategy in HCC crucially needs improved stratification to better define the population who could benefit from adjuvant treatment from the population who does not need further therapeutic intervention. Considering the results of the present study and the preceding considerations, new trials directed to patients with a higher risk of relapse than the STORM population might be worthwhile to redeem the effect of sorafenib as adjuvant treatment. Nevertheless, improved staging combining histological and biological parameters would be an important step forward discriminating subgroups prognosis and therapeutic interventions.

Finally, in the present time of exploding medical costs, adjuvant trials should consider the cost-effectiveness of long-term treatments, which emphasize the need of better stratifying patients to focus on the appropriate population [5]. As hypothesized in the STORM trial, a 30% increase in recurrence-free survival may be worth the cost of several years of an expensive drug before it goes generic. Moreover, when positive, such trials could also lead to follow-up studies better defining the targeted patient populations. Considering the intrinsic medical and biological differences between adjuvant and advanced settings, it may be time to consider new tools and new arguments to develop drugs in adjuvant settings.

## Abbreviations

HCC: Hepatocellular carcinoma
STORM: Sorafenib as adjuvant treatment in the prevention of recurrence of hepatocellular carcinoma
